# Combination Therapy of Sorafenib and TACE for Unresectable HCC: A Systematic Review and Meta-Analysis

**DOI:** 10.1371/journal.pone.0091124

**Published:** 2014-03-20

**Authors:** Lei Liu, Hui Chen, Mengmeng Wang, Yan Zhao, Guohong Cai, Xingshun Qi, Guohong Han

**Affiliations:** 1 Department of Digestive Interventional Radiology, Xijing Hospital of Digestive Diseases, Xijing Hospital, Fourth Military Medical University, Xi'an, Shaanxi, China; 2 Department of Gastroenterology, First Affiliated Hospital of the Medical College, Xi'an Jiaotong University, Xi'an, Shaanxi, China; 3 Department of Anatomy, Histology and Embryology, K.K. Leung Brain Research Centre, Fourth Military Medical University, Xi'an, China; 4 Department of Health Services, School of Military Preventive Medicine, Fourth Military Medical University, Xi'an, China; Northwestern University Feinberg School of Medicine, United States of America

## Abstract

**Background and Aim:**

A large number of studies have tried to combine sorafenib with TACE for patients with unresectable hepatocellular carcinoma (HCC) and the results were controversial. We conducted this systematic review and meta-analysis to evaluate the safety and efficacy of combination therapy of sorafenib and TACE in the management of unresectable HCC.

**Methods:**

MEDLINE, PsycINFO, Scopus, EMBASE, and the Cochrane Library were searched from January 1990 to October 2013 and these databases were searched for appropriate studies combining TACE and sorafenib in treatment of HCC. Two authors independently reviewed the databases and extracted the data and disagreements were resolved by discussion. Effective value and safety were analyzed. Effective value included disease control rate (DCR), time to progression (TTP) and overall survival (OS).

**Results:**

17 studies were included in the study. In the 10 noncomparative studies, DCR ranged from 18.4 to 91.2%. Median TTP ranged from 7.1 to 9.0 months, and median OS ranged from 12 to 27 months. In the 7 comparative studies, the hazard ratio (HR) for TTP was found to be 0.76 (95% CI 0.66–0.89; P<0.001) with low heterogeneity among studies (P = 0.243; I^2^ = 25.5%). However, the HR for OS was found to be 0.81 (95% CI 0.65–1.01; P = 0.061) with low heterogeneity among studies (P = 0.259; I^2^ = 25.4%). The common toxicities included fatigue, diarrhea, nausea, hand foot skin reaction (HFSR), hematological events, hepatotoxicity, alopecia, hepatotoxicity, hypertension and rash/desquamation. AEs are generally manageable with dose reductions.

**Conclusions:**

Combination therapy may bring benefits for unresectable HCC patients in terms of TTP but not OS. Further well-designed randomized controlled studies are needed to confirm the efficacy of combination therapy.

## Introduction

Hepatocellular carcinoma (HCC) is the fifth most common malignancy worldwide and the third most common cause of cancer-related deaths [1]. The incidence rates are highest in East Asia, sub-Saharan Africa and Melanesia, and it is also increasing in Europe and the United States [2], [3].

The Barcelona Clinic Liver Cancer (BCLC) staging system is the most widely used system advocated for prognostic classification of HCC all over the world, which has been recommended by the European Association for the Study of the Liver (EASL) and the American Association for the Study of Liver Diseases (AASLD) [4], [5]. It combines information on prognostic prediction and treatment allocation. For instance, transarterial chemoembolization (TACE) is recommended as the standard of care for patients with intermediate stage HCC (BCLC stage B), who are with large or multinodular HCC while without portal vein tumor thrombosis (PVTT) or extrahepatic metastasis. In addition, sorafenib, as a multikinase inhibitor, is the current standard therapy for advanced HCC (BCLC stage C), which is characterized by an Eastern Cooperative Oncology Group (ECOG) performance status of 1–2 and/or the presence of PVTT or extrahepatic metastasis [6].

However, the high rate of tumor recurrence and low rate of long-term survival are still common in patients with unresectable HCC, making more effective and safer therapies to be urgently needed [7]. TACE is a locoregional therapy and could embolize the hepatic artery. Molecular biology studies have shown that the level of vascular endothelial growth factor (VEGF) usually increases locally and systemically after TACE treatment is performed, whereas sorafenib can inhibit the activity of VEGF receptors [8], [9]. Thus, in recent years a large amount of studies have tried to combine sorafenib with TACE for patients with unresectable HCC, while the results were controversial [10]–[27]. Currently it remains unknown whether combination therapy could improve the survival of patients.

Therefore, we conducted the systematic review and meta-analysis to evaluate the outcomes of sorafenib in combination with TACE in treating unresectable HCC patients.

## Methods

### Identification and Eligibility of Relevant Studies

**Figure 1:**
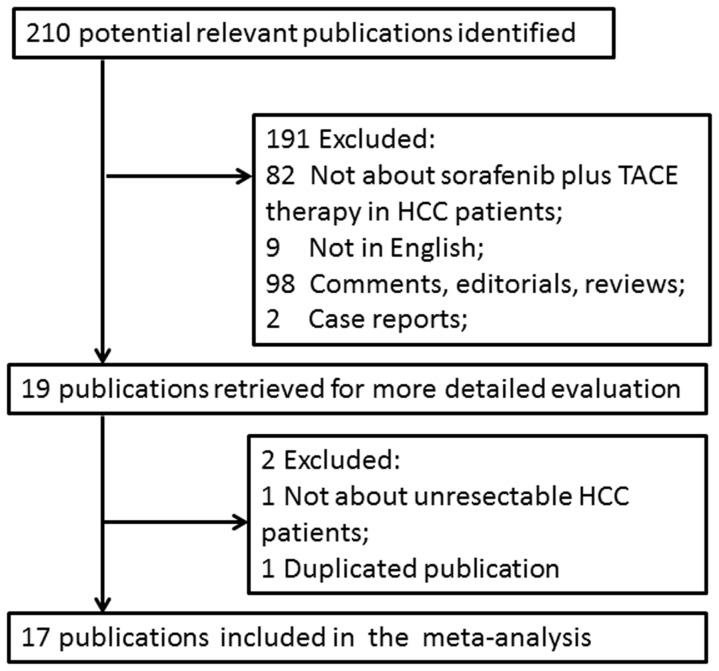
Flow diagram of the study selection procedure.

We considered all studies examining the efficacy and safety of combination therapy of sorafenib with TACE for the management of unresectable HCC patients. The electronic databases screened were MEDLINE (January 1990 to October 2013), PsycINFO (January 1990 to October 2013), Scopus (January 1990 to October 2013), EMBASE (January 1990 to October 2013), and the Cochrane Library (Issue 10 of 12, October 2013). The search terms were: “transarterial chemoembolization” or “TACE” or “chemoembolization” AND “hepatocellular carcinoma” or “HCC” or “liver cancer” or “liver tumor” or “hematoma” AND “sorafenib”. Searches were limited to original articles in English and performed for all types of publications. We also screened the references of retrieved articles and contacted with the authors for additional data when key information relevant to the meta-analysis was missing. The flow chart of the systematic review and meta-analysis was shown in Figure 1, which was developed from PubMed and was adapted for the other electronic databases.

### Inclusion Criteria

1. Clinical trials that described sorafenib in combination with TACE in treating advanced/unresectable HCC patients.
2. Only adults were included in the Clinical trial.
3. TTP, OS, Tumor response outcome measures or toxicities were reported in these articles.

### Exclusion Criteria

1. Clinical trials that described the clinical value of sorafenib in combination with TACE in patients with unresectable HCC compared with efficacy of sorafenib alone.
2. Non English language records were also excluded.

### Definitions and Standardizations

#### TACE

The data included in the literature review related to conventional TACE (cTACE) and TACE with drug-eluting beads (DEB-TACE). A recent systematic review had collected sufficient data on the use of DEB-TACE in HCC patients to support its use as a safe and effective chemo-embolic treatment in intermediate HCC patients, however, there still needs more strong evidence to support the its superiority over c-TACE [28].

#### Sorafenib

Sorafenib is the first FDA-approved systemic therapy for patients with advanced HCC. Nevertheless, the administration schedules of sorafenib are still varied. In the current data, sorafenib was given before the first TACE session in some studies, while in other studies the administration of sorafenib started after TACE performance.

#### Comparative study

The comparative studies included in our study were all about clinical value of sorafenib in combination with TACE in patients with unresectable HCC compared with efficacy of TACE alone.

#### Disease control rate

Complete response rate + partial response rate + stable disease rate.

### Data Extraction

Two of us (L.L., H.C.) independently screened the titles and abstracts of potentially eligible studies, and then examined the full text articles to determine whether they met the inclusion criteria. Meanwhile, they kept a record of reasons for excluding studies. Three of us (Y.Z., G.C., and M.W.) independently extracted data (study characteristics and results) using data extraction forms, and then the collected data were put into STATA 12.0. When it came to a disagreement between the two reviewers, a consensus was achieved through discussion among all of the reviewers.

### Data Collection

We collected the following data and information:

1. General study information such as title, publication year, authors, country, and type of study.
2. Characteristics of the study population (e.g. number of patients, Child-Pugh Score, performance status, classification of HCC and Hepatitis).
3. Characteristics of the treatment, containing the administration schedules of sorafenib and TACE.
4. Characteristics of treatment efficacy, such as disease control rate, time to progression and overall survival.
5. Summary of toxicities of treatment.

### Data Analysis

Meta-analyses were conducted using STATA 12.0 according to Cochrane Handbook for Systematic Reviews of Interventions [29]. Data on time to progression and overall survival were combined across studies using hazard ratio (HR). Measurements from the graph were used if we could not get the data from the authors. I^2^ statistics were used to measure heterogeneity of the studies. If the I^2^ value was less than 50%, a fixed-effects meta-analysis was applied. If the I^2^ value was 50% or more, the random-effects meta-analysis was performed [29].

## Results

The flow chart of our study was shown in Figure 1. Finally, 17 studies met our selection criteria and were included in this study. The detailed characteristics of included noncomparative and comparative studies were described in Table 1 and 2 [10]–[19], [21]–[27]. A total of 676 patients with unresectable HCC were included in the 10 noncomparative studies (Table 1), which contained 6 phase-II studies, 2 phase-I studies and 2 retrospective studies. The number of patients from individual studies ranged from 14 to 222. All the patients had Child-Pugh (CP) class A or B severity of disease, and the frequency of CP-A patients ranged from 65% to 94%, indicating well compensated disease. The vast majority (84–100%) of patients was in BCLC B or C stage and most of their ECOG Performance Status was reported to be 0 or 1 (94–100%). 9 studies described etiology of the patients, and the total incidence of viral hepatitis ranged from 24% to 100%. TACE was conventional in 8 studies, and used drug-eluting beads (DEB) in 2 studies. The median/mean number of TACE sessions ranged from 1 to 3.

**Table 1:** Baseline characteristics of noncomparative studies assessing effect of combination therapy for unresectable HCC.

Authors (year)	Study Design	Country	Pts	CPS	BCLC	ECOG	Hepatitis	Treatment characteristics	NO. of TACE
Erhardt *et al.* (2009) [10]	Phase II	Germany	38	≤8 scores	NA	0–2	NA	Continuous sorafenib (400 mg bid), interrupted only around TACE	2.0(mean)
Dufour *et al.* (2010) [11]	Phase I, Open-label	Switzerland	14	A = 93%;B = 7%	B = 64% C = 36%	0 = 93% 1 = 7%	HCV = 29%	Sorafenib started 1 week prior to TACE without a pause for TACE	2.0(median)
Cabrera *et al.* (2011)† [13]	Phase II Prospective	USA	47	A = 72%;B = 28%	B = 81% C = 19%	0 = 75% 1 = 25%	HCV = 60%	Continuous sorafenib started 2–4 weeks before TACE	3.0(median)
Lee *et al.* (2011) [12]	Phase II Prospective	South Korea	59	A = 93%;B = 7%	B = 100%	NA	HBV = 88%	Sorafenib (400 mg bid), TACE was performed at every 6–8 weeks	NA
Pawlik *et al.* (2011)† [14]	Phase II Prospective	USA	35	A = 89%;B = 11%	B = 34% C = 66%	0 = 46% 1 = 54%	HCV = 37%	Continuous sorafenib (400 mg bid) started 1 week before DEB-TACE	2.0(median)
Chung *et al.* (2012) [15]	Phase II Prospective	China and South Korea	151	A = 92% B = 8%	A = 16% B = 82% C = 1.9%	0 = 82% 1 = 18%	NA	Sorafenib(400 mg bid) started 4–7 days after TACE	2.1(mean)
Park *et al.* (2012) [16]	Phase II Prospective	South Korea	50	A = 94% B = 6%	B = 82% C = 18%	0 = 44% 1 = 56%	HBV = 68% HCV = 18%	Sorafenib was given 3 days after TACE and was administered for up to 24 weeks	1.0(median)
Qu *et al.* (2012) [17]	Retrospective	China	45	A = 65% B = 35%	B = 35% C = 65%	0 = 95% 1 = 5%	HBV = 100%	Sorafenib (400 mg bid) started after TACE, the duration time of taking sorafenib is 11.61±5.3 months	NA
Sieghart *et al.* (2012) [18]	Phase I	Austria	15	A = 80% B = 20%	B = 70% C = 30%	0 = 92% 1 = 8%	HBV = 4% HCV = 20%	Sorafenib (400 mg bid) started 2 weeks before the first TACE. Median time on sorafenib was 5.2 months.	3.0(median)
Zhao *et al.* (2013) [19]	Retrospective	China	222	A = 86% B = 14%	B = 20% C = 80%	0 = 44% 1 = 50% 2 = 6%	HBV = 80% HCV = 5%	Continuous sorafenib (400 mg bid) with no breaks before or after TACE	2.0(median)

**Note:**

† †: TACE was performed using the drug-eluting beads (DEB) in the study. Other studies were conventional TACE.

**Abbreviations:** BCLC, The Barcelona Clinic Liver Cancer; CPS, Child-Pugh classification; ECOG, Eastern Cooperative Oncology Group; NO., number; HBV, hepatitis B virus; HCV, hepatitis C virus; HCC, Hepatocellular carcinoma; NA: not available; Pts., patients; TACE, transarterial chemoembolization.

**Table 2:** Baseline characteristics of comparative studies assessing effect of combination therapy for unresectable HCC.

Authors (year)	Study Design	Country	Pts.	CPS	BCLC	ECOG	Hepatitis	Treatment characteristics	NO. of TACE
Martin *et al.* 2010† [21]	Prospective	several countries	150	ST: B = 31% DT:B = 39%	NA	NA	NA	ST(n = 30) versus DT (n = 120)	ST: 2.0; DT: 1.0 (median)
Kudo *et al.* 2011 [22]	Phase III, Randomized	Japan and South Korea	299	A = 100%	NA	0 = 87% 1 = 13%	HBV = 20% HCV = 60%	Sorafenib was given 1–3 months after TACE till progression	1.0–2.0
Sansonno *et al.* 2012 [24]	phase II, prospective, randomized	Italy	40	A = 100%	B = 100%	0 = 86% 1 = 24%	HCV = 100%	Sorafenib started 30 days after TACE till progression or unacceptable toxicity	NA
Lencioni *et al.* 2012† [23]	phase II, prospective, randomized	several countries	307	A = 100%	B = 100%	0 = 100%	NA	Continuous sorafenib 3–7 d before chemoembolization	NA
Bai *et al.* 2013 [25]	Prospective	China	82	A = 77% B = 23%	B = 23% C = 77%	0 = 36.5%, 1 = 46.5%, 2 = 14.6%, 3 = 1.2%, 4 = 1.2%	HBV = 87% HCV = 5%	Continuous sorafenib (400 mg orally bid), initiated within 14 days after TACE	NA
Muhammad *et al.* 2013† [26]	Retrospective	USA	43	ST:A = 85%DT:A = 77%	A = 46%; B = 15%; C = 38%	NA	ST:HCV = 69% DT:HCV = 93%	Sorafenib started with 200 mg bid and then increased to 400 mg in the majority of patients	1.9 (mean)
Huang *et al.* 2013 [27]	Prospective	China	155	NA	NA	NA	NA	Sorafenib started within 2 weeks of the first cycle of TACE	NA

**Note:**

† †: TACE was performed using the drug-eluting beads (DEB) in the study. Other studies were conventional TACE.

**Abbreviations:** BCLC, The Barcelona Clinic Liver Cancer; CPS, Child-Pugh classification; Pts., patients; ST: sorafenib+TACE;DT: DEB-TACE; ECOG, Eastern Cooperative Oncology Group; HBV, hepatitis B virus; HCV, hepatitis C virus; HCC, Hepatocellular carcinoma; NA: not available; TACE, transarterial chemoembolization.

In the 7 comparative studies (Table 2), which contained 3 randomized controlled studies, 3 nonrandomized contrast studies and 1 contrast retrospective studies, a total of 1076 patients with unresectable HCC were included. The number of patients from individual studies ranged from 40 to 307. The vast majority (77–100%) of patients had Child-Pugh class A severity of disease, also indicating well compensated disease. Most of the patients were in BCLC B or C stage and their ECOG Performance Status was 0 or 1 (83–100%). The proportion of patients with underlying HBV and HCV varied considerably between the 7 studies. Conventional TACE was performed in 4 studies and DEB-TACE in 3 studies. The median/mean number of TACE sessions ranged from 1 to 2.

### Tumor Response, TTP and OS

In the 10 noncomparative studies, 4 studies used modified RECIST (response evaluation in solid tumors) to assess tumor response, 4 studies used RECIST, and the remaining 2 studies did not report. The disease control rate (DCR) was reported ranging from 18.4 to 91.2%. Most of the DCR was reported to be around 80%, while much lower DCR (68% and 18.4%) was shown by Cabrera et al. and Erhardt et al. Interestingly, in the study reported by Zhao et al., only 2% of the patients experienced a complete response (CR) according to RECIST criteria, while 27% of the patients achieved CR according to modified RECIST [19].

Median TTP was reported in five out of the 10 noncomparative studies and there was inter-trial variability in median progress free survival (PFS) (Table 3), which ranged from 7.1 to 9.0 months. Median OS was reported in 3 noncomparative studies, ranging from 12 to27 months (Table 3).

**Table 3:** Tumor Response, TTP and OS in the 10 noncomparative studies.

Author (year)[Ref]	Response criteria	DCR (%)	Median TTP (months)	Median OS (month)
Erhardt *et al.* [10]	RECIST	18.4	NA	NA
Dufour *et al.* [11]	NA	NA	NA	NA
Cabrera *et al.* [13]	Modified RECIST	68	NA	18.5 (95% CI 16.1–20.9)
Lee *et al.* [12]	Modified RECIST	76	NA	NA
Pawlik *et al.* [14]	RECIST	95	NA	NA
Chung *et al.* [15]	Modified RECIST	91.2	9	NA
Park *et al.* [16]	RECIST	84	7.1 (95% CI, 4.8–7.5)	NA
Qu *et al.* [17]	NA	NA	NA	27 (95% CI 21.9–32.1)
Sieghart *et al.* [18]	Modified RECIST	80	NA	NA
Zhao *et al.* [19]	RECIST	86	NA	12 (95% CI 10.1–13.9)

**Abbreviations:** DCR, disease control rate; NA: not available; OS, overall survival; RECIST, response evaluation in solid tumors; TTP, time to progression.

**Figure 2:**
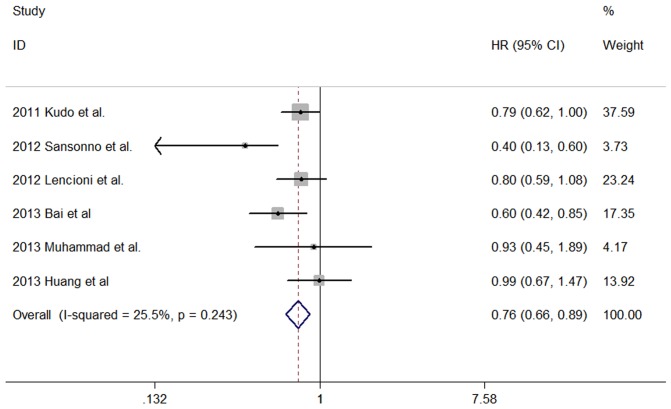
Forest plot showing the associations of the time to progression (TTP) between TACE alone group and sorafenib combined with TACE group for patients with unresctable HCC. The result of meta-analysis for TTP between TACE alone group and sorafenib combined with TACE group for patients with unresctable HCC. Studies are arranged by publication year. Forrest plot displayed as hazard ratio and 95% confidence intervals. (HR, hazard ratio; CI, confidence interval).

**Figure 3:**
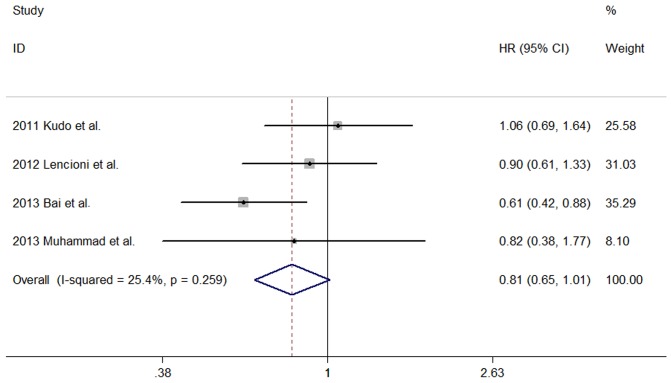
Forest plot showing the associations of the overall survival (OS) between TACE alone group and sorafenib combined with TACE group for patients with unresctable HCC. The result of meta-analysis for OS between TACE alone group and sorafenib combined with TACE group for patients with unresctable HCC.Studies are arranged by publication year. Forrest plot displayed as hazard ratio and 95% confidence intervals. (HR, hazard ratio; CI, confidence interval).

In the included comparative studies, 6 studies [22]–[27] presented available data of the hazard ratio (HR) for TTP and 4 studies [22], [23], [25], [26] presented available data of the HR for OS. The HR for TTP was found to be 0.76 (95% CI 0.66–0.89; P<0.001) with low heterogeneity among studies (P = 0.243; I2 = 25.5%) (Figure 2), suggesting the combined use of sorafenib and TACE may improve TTP compared with TACE alone in patients with unresctable HCC. However, the HR for OS was found to be 0.81 (95% CI 0.65–1.01; P = 0.061) with low heterogeneity among studies (P = 0.259; I2 = 25.4%), indicating the combined use of sorafenib plus TACE might not improve OS compared with TACE alone in patients with unresctable HCC (Figure 3).

### Adverse events

The adverse events (AEs) experienced during combination therapy in 10 noncomparative studies were shown in Table 4. The more common toxicities included fatigue, diarrhea, nausea, hand foot skin reaction (HFSR), hematological events, hepatotoxicity, alopecia, hepatotoxicity, hypertension and rash/desquamation, which mostly were grade 1 or 2. Noted toxicity-related reasons for treatment discontinuation included hepatotoxicity, HFSR and diarrhea; however, detailed reasons for patient withdrawal were unknown in all studies. AEs are generally manageable with dose reductions [13].

**Table 4:** The adverse events experienced during combination therapy in 10 noncomparative studies.

Author[Ref]	Fatigue (%)	Diarrhea (%)	HFSR (%)	Hematological events (%)	Alopecia (%)	Hepatotoxicity (%)	Hypertension (%)	Nausea (%)	Rash/Desquamation (%)
Erhardt *et al.* [10]	5	NA	5	NA	NA	5	NA	NA	NA
Dufour *et al.* [11]	NA	50	21	14	NA	20	NA	NA	NA
Cabrera *et al.* [13]	51	42.5	51.1	10.6	4.3	23	19	14.9	15
Lee *et al.* [12]	NA	NA	NA	NA	NA	NA	NA	NA	NA
Pawlik *et al.* [14]	80	33	40	38	35	55	NA	NA	NA
Chung *et al.* [15]	8.2	18.4	3.4	6.8	25.9	4	8.8	19	28
Park *et al.* [16]	10	48	74	14	24	36	NA	54	12
Qu *et al.* [17]	47	37.8	54	81	37.8	NA	33.3	24	42.2
Sieghart *et al.* [18]	95	50	4	50	75	75	17	50	20
Zhao *et al.* [19]	33	50	44	NA	NA	4	NA	3	39

**Abbreviations:** HFSR, hand foot skin reaction; NA, not available.

## Discussion

The present systematic review and meta-analysis provides comprehensive data about the combination therapy for unresectable HCC patients. In all comparative studies, the efficacy and safety was compared between the patients in the study group receiving sorafenib plus TACE treatment and the patients in the control group receiving TACE alone. The meta-analysis showed that sorafenib combined with TACE may have superiority over TACE alone in terms of TTP but not OS.

We included 3 randomized controlled trials in this systematic review and meta-analysis. Only 1 study by Sansonno et al. demonstrated that conventional TACE followed by sorafenib treatment resulted in a significantly longer TTP in patients with intermediate stage HCC. However, the sample size was small (31 versus 31), which limited the evidence level [24]. In addition, the SPACE trial was the first global randomized controlled trial with a large sample size (154 versus 153) to explore the superiority of combination therapy over TACE alone in intermediate stage HCC patients [23]. However, the results of SPACE trial were just passable. The difference of TTP, as the primary endpoint, was not statistically significant between two groups. We consider that the reason lies in this trial's design. In this trial, TACE treatments were performed on-schedule. More than one-third of patients received only one session of TACE in the study group, which limited the efficacy of TACE for controlling the progression of local tumor lesions. Because repetition of TACE is based on evidence suggesting that one cycle of TACE may not be sufficient for effective treatment of intermediate-stage HCC [30], [31].

In addition, the study by Bai et al. was a prospective non-randomized controlled trial with the sample size 82 versus 164 [25]. This study reported positive results that combination therapy improved both OS and TTP compared with TACE alone in patients with unresectable HCC. Firstly, the different study design from SPACE trial was that in this study the TACE was performed when residual viable tumors were confirmed or new lesions developed. Secondly, the incidence of dose reduction of sorafenib and dose interruption were also lower than that in SPACE trial (9.8% versus 87.1%, 19.5% versus 84.1%). It was reported that sorafenib interruption may cause tumor rebound because the VEGF will increase sharply following TACE performance [11]. Thirdly, in this study both BCLC-C stage patients were also included. Therefore, these points mentioned above may contribute to the positive outcomes in terms of OS and TTP. However, the nonrandomized nature was the potential limitations of this study.

Although the meta-analysis of this review was negative on the result of OS, the combination of sorafenib and TACE is still very hopeful in improving the survival time in selected HCC patients. A recent study showed that TACE plus sorafenib was superior to sorafenib alone with respect to TTP in patients with advanced-stage HCC, although it may or may not improve OS [32]. We consider that the issue is which patients may benefit from treatment and which schedule of TACE should be applied. Especially selection of candidates for combination therapy is a key point. In order to achieve the best outcomes, there must be careful selection of patients for combination therapy and a reasonable study design to yield the greatest efficacy of TACE. Several phase III trials evaluating sorafenib with TACE are ongoing and may resolve some of these outstanding issues with regard to combination therapy [33].

This study is accompanied with the following limitations. First, although the heterogeneity of available data from these studies was not obvious, there are many elements we should take into consideration, such as the number of patients, BCLC stage, and treatment characteristics et al. Second, only 4 studies included in the OS analysis and only 6 studies included in the TTP analysis because other studies are noncomparative and no detailed information was available. Third, some methods used in this article are limited, such as using I2 for assessing the amount of heterogeneity in random-effects meta-analysis.

In conclusion, this systematic review and meta-analysis demonstrated that sorafenib combined with TACE may have superiority over TACE alone in terms of TTP but not OS. Further well-designed randomized controlled studies are needed to evaluate whether combination therapy has superiority over TACE treatment alone in terms of overall survival. Selecting adequate target population, applying reasonable approach of combination and TACE schedule are essential for obtaining positive outcomes.

## Supporting Information

Checklist S1 **PRISMA Checklist.**
(PDF)
